# Design of an Intelligent Decision Support System Applied to the Diagnosis of Obstructive Sleep Apnea

**DOI:** 10.3390/diagnostics13111854

**Published:** 2023-05-25

**Authors:** Manuel Casal-Guisande, Laura Ceide-Sandoval, Mar Mosteiro-Añón, María Torres-Durán, Jorge Cerqueiro-Pequeño, José-Benito Bouza-Rodríguez, Alberto Fernández-Villar, Alberto Comesaña-Campos

**Affiliations:** 1 Department of Design in Engineering, University of Vigo, 36208 Vigo, Spain; jcerquei@uvigo.es (J.C.-P.); jbouza@uvigo.es (J.-B.B.-R.); acomesana@uvigo.es (A.C.-C.); 2 Design, Expert Systems and Artificial Intelligent Solutions Group (DESAINS), Galicia Sur Health Research Institute (IIS Galicia Sur), SERGAS-UVIGO, 36213 Vigo, Spain; 3 Pulmonary Department, Hospital Álvaro Cunqueiro, 36213 Vigo, Spain; maria.luisa.torres.duran@sergas.es (M.T.-D.); alberto.fernandez.villar@sergas.es (A.F.-V.); 4 NeumoVigo I+i Research Group, Galicia Sur Health Research Institute (IIS Galicia Sur), SERGAS-UVIGO, 36213 Vigo, Spain

**Keywords:** obstructive sleep apnea, design, clinical decision support system, intelligent system, expert system, machine learning, decision making, medical algorithm, design science research

## Abstract

Obstructive sleep apnea (OSA), characterized by recurrent episodes of partial or total obstruction of the upper airway during sleep, is currently one of the respiratory pathologies with the highest incidence worldwide. This situation has led to an increase in the demand for medical appointments and specific diagnostic studies, resulting in long waiting lists, with all the health consequences that this entails for the affected patients. In this context, this paper proposes the design and development of a novel intelligent decision support system applied to the diagnosis of OSA, aiming to identify patients suspected of suffering from the pathology. For this purpose, two sets of heterogeneous information are considered. The first one includes objective data related to the patient’s health profile, with information usually available in electronic health records (anthropometric information, habits, diagnosed conditions and prescribed treatments). The second type includes subjective data related to the specific OSA symptomatology reported by the patient in a specific interview. For the processing of this information, a machine-learning classification algorithm and a set of fuzzy expert systems arranged in cascade are used, obtaining, as a result, two indicators related to the risk of suffering from the disease. Subsequently, by interpreting both risk indicators, it will be possible to determine the severity of the patients’ condition and to generate alerts. For the initial tests, a software artifact was built using a dataset with 4400 patients from the Álvaro Cunqueiro Hospital (Vigo, Galicia, Spain). The preliminary results obtained are promising and demonstrate the potential usefulness of this type of tool in the diagnosis of OSA.

## 1. Introduction

Obstructive sleep apnea (OSA) is a chronic disease characterized by episodes of total or partial collapse of the upper airway during sleep, which impairs its quality and causes daytime sleepiness and fatigue. In addition to these, if left untreated, OSA has a direct impact on the patient’s health as it can cause hypertension and an increased risk of cardiovascular and cerebrovascular accidents, as well as being associated with the development of cognitive and metabolic disorders, among others.

Faced with this problem, and with approximately one thousand million people worldwide suffering from OSA [1], efforts have been made in the most developed countries to diagnose OSA at an early stage and to treat patients with this pathology. In 2015, OSA-related expenditure in United States was 12.4 thousand million dollars [2,3]. Despite this, it has been observed that a large number of patients suffering from this condition remain undiagnosed and therefore untreated [1,4], a fact that cannot be ignored due to the high health impact of this disease.

In-lab polysomnography is currently the gold standard for diagnosing OSA [5,6,7,8,9,10] and consists of a series of physiological measurements during sleep that allow the characterization of the pathology. However, there are other alternatives for diagnosing OSA that are cheaper and simpler. One of these is cardiorespiratory polygraphy; however, this does not provide information on neurophysiological variables [11,12]. After these studies, the apnea-hypopnea index (AHI) is the most commonly used variable to describe and assess the pathology. It measures the number of apnea (a complete interruption of respiratory function for at least 10 s [13,14]) and hypopnea (a decrease of at least 30% in respiratory flow for at least 10 s and a microarousal or desaturation less than 4% [13,14]) events that a patient experiments in an overninght sleep study divided by the total hours slept [8,15].

Although in-lab polysomnography is a widely used and recognized technique, it cannot be used for mass screening of the general population, at least with the technology currently available, because of its complexity and associated high cost [8,9]. It should be also noted that the number of accredited centers with this type of equipment is limited, which means that many patients are only referred to this type of study when they present with severe symptoms after a long period of suffering from the pathology [9]. All this points to the need for standardized methods to improve the screening process, thereby reducing the number of patients who are referred to the sleep units. Thus, priority would be given to those patients in need of it, which would result in an improvement in the diagnostic process and a decrease in the associated costs.

In this context, and in view of the problem described, this work deals with the design and development of a novel intelligent decision support system for the diagnosis of patients suspected of suffering from OSA. To this end, heterogeneous patient information is considered, both quantitative (age, body mass index, neck circumference, diagnosed conditions and prescribed treatments) and qualitative (symptoms reported by the patient in a sleep interview). From that, the intelligent system will be able to determine, through the concurrent [16,17,18,19,20,21,22] use of a machine-learning classification algorithm and a cascade of expert systems based on the Mamdani-type fuzzy inference system [23,24,25,26], two indicators associated with the risk of suffering from OSA. The first one is related to data of a more objective nature, while the second one is associated with those of a more subjective nature. Next, both risk indicators are evaluated, allowing us to determine if the patient is at risk of suffering from the disease, which would then require further confirmatory diagnostic studies to be performed.

This paper is structured into five sections. The remainder of Section 1 discusses the use of artificial intelligence approaches for the diagnosis of OSA. In Section 2, the conceptual description of the proposed system is presented, explaining the different stages involved, as well as the information flow. Then, the implementation of the system is detailed. Section 3 presents the results obtained from the case study. Next, Section 4 discusses the proposed system, and finally, Section 5 presents the conclusions and future lines of development.

### Artificial Intelligence Approaches for the Diagnosis of Obstructive Sleep Apnea

It is becoming increasingly common in the healthcare field to have tools and approaches to support decision-making processes [27,28,29,30,31,32,33,34,35,36]. In particular, given the complexity of the OSA diagnostic process, several specific tools have been developed and proposed in recent years to support the diagnostic process.

In the work by Corrado Mencar et al. [37], the efficacy and applicability of machine-learning approaches were analyzed on a dataset with 313 patients from two sleep units in Italy. Both demographic data and questionnaires were used to determine the degree of severity of the patient’s OSA. Classification approaches were used, with support vector machines and random forest achieving the best results, with a maximum accuracy of 44.7% in the test set. Regression approaches were used to determinate the AHI level, with the best results obtained with support vector machines and linear regression, which had a minimum root mean square error value of 22.17.

Along this line, in the work by Lei Ming Sun et al. [38], based on data collected from questionnaires of 110 suspected patients who performed a polysomnography in the teaching hospital in Taiwan, an approach was proposed that seeks to screen those patients with moderate-severe OSA (with an AHI ≥ 15). For this purpose, genetic algorithms were implemented, obtaining a sensitivity of 81.8% and an accuracy of 88.4% for the test set. On the other hand, logistic regression showed a sensitivity of 55.6% and an accuracy of 57.2%. The authors report that the prevalence of apnea in their dataset was 77%, which is far from the real situation (common prevalence is 2 to 8% in general population [38]), so the model may have problems when extended to real populations.

In the work by Jayroop Ramesh et al. [39], the use of machine-learning approaches was proposed to discriminate between patients suspected of suffering from OSA, establishing an AHI threshold value of 5. To achieve this, the Wisconsin Sleep Cohort dataset with a total of 1479 patients (which included demographic information, physical measurements of the patient or sleep history, among other possible questions) was used. Firstly, feature selection techniques were applied to reduce the number of predictors. After applying optimization techniques and training different models, it was observed that the use of support vector machines was the model that showed the greatest results, with an accuracy of 68.06% and a sensitivity of 88.76%.

In the work by Daniela Ferreira-Santos and Pedro Pereira-Rodrigues [40], the use of Bayesian network classifiers, more specifically naïve Bayes and tree augmented naïve Bayes, was proposed to help distinguish between patients who may suffer from OSA in order to be able to decide which of them need to undergo polysomnography. With this aim, using data from 194 patients, two possible situations had to be considered. In the first one, the models were built with 38 variables, and accuracies of 67.1% and 66.9% and sensitivities of 90.0% and 81.9% were observed for the naïve Bayes and tree augmented naïve Bayes models, respectively. The second scenario, which considered only a selection of six variables based on a body of knowledge review, showed accuracies of 70.2% and 67.5% and sensitivities of 94.1% and 90.2% for the naïve Bayes and tree augmented naïve Bayes models, respectively.

In the work by C. Zoroglu and S. Turkeli [41], an expert system based on a Mamdani-type fuzzy inference system was proposed, which used the body mass index, the minimum blood oxygen saturation during sleep, the Mallampati score and the neck diameter to infer a level of AHI related to the risk of suffering from OSA.

Similarly, in the work by J. M. Matthews et al. [42] and based on the responses to the STOP-Bang questionnaire, a fuzzy rule-based system for the screening of OSA patients was presented.

It is also important to note the authors’ previous work in the field of OSA, published recently in 2023 [21], in which they presented the architecture of a then-novel intelligent decision support system. To this end, based on the information related to the patient’s health profile (the objective information mentioned above), the use of a series of machine-learning algorithms that work concurrently was proposed, focused on different AHI levels (10, 15, 20, 20, 25 and 30), with the aim of discriminating between different degrees of severity of the condition. The system also included a corrective block, based on the use of adaptive neuro-fuzzy inference system (ANFIS) and a particular heuristic algorithm, through which it was possible to correct anomalous or undesired behaviors. The initial tests of the system were carried out using a database from the Álvaro Cunqueiro Hospital (Vigo, Galicia, Spain), obtaining results that were supported by values of the Matthews correlation coefficient close to 0.6.

It may be appreciated that the analyzed works mostly use artificial intelligence approaches that implement learning models, thus requiring a dataset on which the algorithm can be trained. However, in the field of OSA, it is not so common to have public (or even private) databases available containing a considerable number of patients. Therefore, it is questionable whether these databases are meaningful and reliable because, generally, the cases in them are limited and do not include different scenarios. This is the reason why the isolated use of learning-based approaches may pose a difficulty when it is desired to build robust and reliable models for clinical diagnosis.

## 2. Materials and Methods

### 2.1. Definition of the System

#### 2.1.1. Database Usage

To conduct this research, a healthcare database from the Respiratory Sleep Disorders Unit of the Pneumology Department of the Hospital Álvaro Cunqueiro (Vigo, Galicia, Spain) was used. This database contains information on 4583 patients, collected between 2013 and 2022. It is important to clarify that the database includes patients who are suspected of having OSA after having been evaluated by specialist pulmonologists, and cannot be considered to represent the general population.

For practical reasons, the database used can be divided into two large groups according to their nature.

On the one hand, there are data showing less subjectivity such as those usually present in electronic health records. For reasons of coherence and to ease the organization of the information, it has been grouped into four categories: general data and anthropometrics (sex, age, weight, height and neck circumference), smoking habits (smokes, does not smoke or smoked in the past, and, if applicable, the number of cigarettes per day and for how many years they have been a smoker) and drinking habits (consumes alcohol regularly, not a consumer or occasional consumer, and, if applicable, the amount of alcohol in grams consumed per day), diagnosed conditions (hypertension, resistant hypertension, acute cerebrovascular accident (ACVA), ACVA within the past year, diabetes mellitus, ischemic heart disease, chronic obstructive pulmonary disease (COPD), home oxygen therapy, rhinitis, depression, atrial fibrillation and heart failure) as well as prescribed treatments (benzodiazepines, antidepressants, neuroleptics, antihistamines, morphics and tranquilizers/hypnotics).

On the other hand, there is information that presents a greater degree of subjectivity related to the symptoms reported by the patient and collected through a specific sleep interview. This is summarized using the following items: hours of sleep, minutes taken to fall asleep, prolonged intra-sleep awakenings, feeling of unrefreshing sleep, daytime tiredness, morning dullness, snorer, high intensity snorer, snoring-related awakenings, unjustified multiple awakenings, nocturia, breathlessness awakenings and reported apneas.

From the initial dataset, 183 patients were randomly selected to be reserved for testing purposes and to illustrate the use of the system in the case study and were thus excluded from the training and validation process of the system. Considering the remaining 4400 patients, after the realization of the sleep studies (in most cases, cardiorespiratory polygraphies) and considering an AHI threshold value of 15, it was observed that 2693 patients presented a value equal or higher than 15, who were then considered as OSA cases, while 1707 presented a lower value and were considered as non-OSA cases. It is important to mention that an AHI value of 15 was chosen because it allows mild OSA cases to be distinguished from moderate–severe ones [43]. However, any other threshold value that the medical team considered appropriate could have been selected.

#### 2.1.2. Conceptual Design and Description of the System

**Figure 1:**
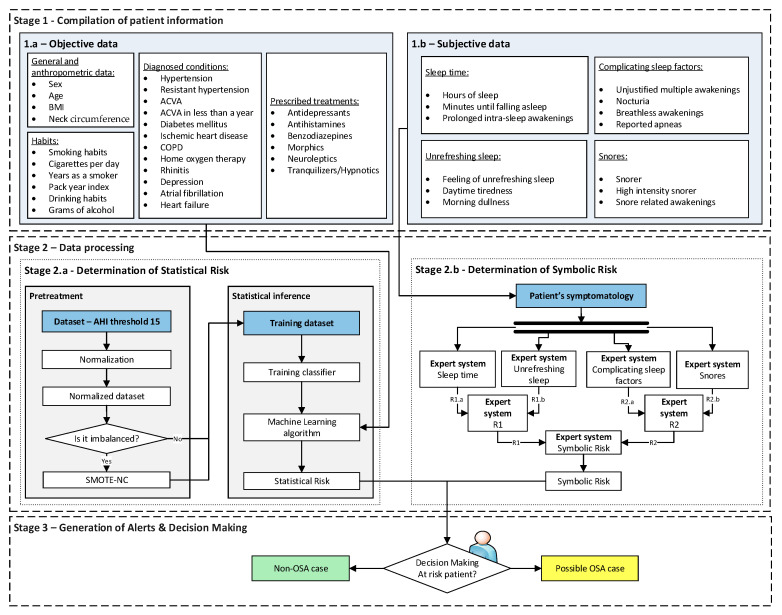
Flow diagram of the intelligent clinical decision support system. The information flow between the different stages that compose the system is shown. Stage 1 is for data collection, Stage 2 is subdivided into Stage 2.a for preprocessing and statistical inference and Stage 2.b for symbolic inference and, finally, Stage 3 is for the generation of alerts and decision making.

Figure 1 shows the flowchart of the proposed intelligent decision support system used to assist in the OSA diagnostic process. A detailed description is presented below.

##### Stage 1: Compilation of Patient Information

The first stage of the proposed intelligent system is focused on the collection of the patient information, which has already been introduced in Section 2.1.1. As already mentioned, and as can be seen in Figure 1, this information can be divided into two main groups depending on the nature of the information:

- Stage 1.a—Objective data: This is associated with a lower degree of subjectivity and interpretability and is summarized in Table 1. This group has been divided into four sub-groups for the sake of coherence and to facilitate the process of entering the data into the forms. Table 1 also indicates whether each data type is numeric or categorical.
- Stage 1.b—Subjective data: This is associated with more interpretative information, related to the symptoms reported by the patient and collected during a sleep interview. This information is summarized in Table 2. As in Table 1, it was decided to divide this group into four sub-groups for the sake of coherence and to simplify their subsequent treatment. Table 2 also includes a description of the type of data, depending on whether it is numerical or categorical.

##### Stage 2: Data Processing

Once the patient information has been collected and structured, it is processed. For this purpose, a machine-learning algorithm and a series of cascaded expert systems are deployed, arranged into two substages that work concurrently [16,17,18,19,20,21,22]. Through those, it is possible to determine two risk indicators, each of them associated with the groups of information previously mentioned, the *Statistical Risk* and the *Symbolic Risk*.

**Figure 2:**
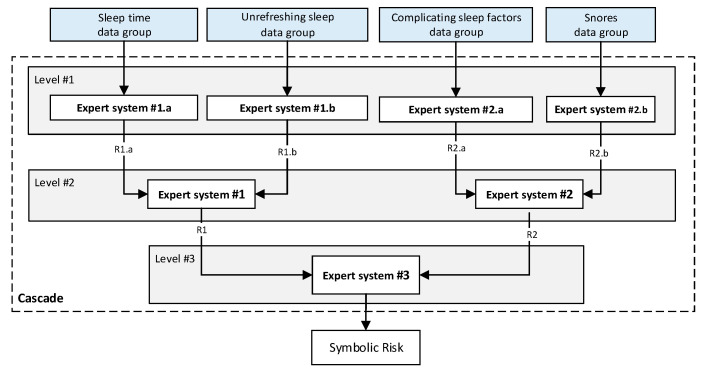
The cascade of expert systems in detail.

- Stage 2.a—Determination of *Statistical Risk*: Once the objective data has been collected, as presented in Stage 1.a, it is processed using a machine-learning classification algorithm [44]. For the definition and configuration of the algorithm a dataset is used, which has already been introduced in Section 2.1.1. This data is preprocessed through normalization and data augmentation approaches, establishing an AHI threshold of 15 to label the different patients according to *OSA case* and *non-OSA case* classes. It is important to note that the medical team could modify this threshold if considered convenient. After this, once the model is adjusted and when new patient data is available, a risk metric will be obtained as the output of the classifier, the *Statistical Risk,* whose value will range from 0 to 100. This indicator can be understood as a percentage risk value of the patient actually suffering from OSA.
- Stage 2.b—Determination of the *Symbolic Risk*: Concurrently to Stage 2.a [16,17,18,19], in Stage 2.b the subjective set of information collected in Stage 1.b is processed. As mentioned above, this information has been divided into four groups. For their processing, a series of expert systems are used, all of them based on the Mamdani-type fuzzy inference system [23,24,25,26] and arranged in a three-level cascade, as shown in Figure 2. This is because it is intended to perform a risk assessment based on different criteria, all of which are involved in the diagnosis of OSA, which allows the reduction of uncertainty and the creation of a more accurate and suitable knowledge base. Nevertheless, since this is a multicriteria approach and the aim is to obtain a global risk indicator that groups and represents them, the risks obtained as an output of the expert systems in the first level of the cascade are simultaneously fuzzified as input to expert systems #1 and #2 in the second level of the cascade. The outputs of these expert systems are also fuzzified as inputs of the expert system #3, which consists of the last level of the cascade, determining as its output a general risk indicator that contemplates the risks of the previous levels. This indicator is called *Symbolic Risk*, and its value will range from 0 to 100, representing the risk associated with the symptoms a patient experiencies as a potential OSA case. It is important to point out that the management of uncertainty in the cascade is not related to probabilities but rather to the concept of fuzzy membership, which is widely known and used in the field of fuzzy logic.

##### Stage 3: Generation of Alerts and Decision Making

Both the *Statistical Risk* and *Symbolic Risk* values obtained in Stage 2 will be initially interpreted individually on the basis of a series of threshold values that allow the establishment of an associated risk level:

- Level 1: This refers to situations in which the level of risk is low, and it seems not to indicate an OSA case. This status will be proposed when the percentage risk to be analyzed is lower than a *Limit 1* value.
- Level 2: This refers to situations in which there is an intermediate level of risk, which does not clearly allow us to distinguish whether or not it is an OSA case. This status will be proposed if the percentage risk value lies in the range [*Limit 1*, *Limit 2*).
- Level 3: This refers to situations in which there is a high risk level that appears to indicate the presence of an OSA case. This status will be proposed when the percentage risk to be analyzed is higher, or equal to, the *Limit 2* value.

Once this has been done, there will be two risk levels, one of them associated with the *Statistical Risk* and the other with the *Symbolic Risk*, and a joint evaluation of these levels will be performed in order to establish a recommendation.

For this purpose, a score will be assigned to each of the levels (a utility function is proposed that transforms the risks into numerical values: for example, if the level is 1, zero points are given; if the level is 2, one point is given; if the level is 3, two points are given). Based on these, a decision variable T will be determined. The expression of this decision variable is shown in Equation (1). In addition, and with the aim of improving the aggregation, a weighting factor has been added to each score, defined through the variable W_ST_ for the statistical score and the variable W_SY_ for the symbolic score. In this regard, $W_{ST}=2-W_{SY}\;$, and $W_{ST}\;and\;W_{SY}\in \left[0,\;2\right]$.

(1) $$T=W_{ST}\cdot Statistical_{score}+W_{SY}\cdot Symbolic_{score}$$

Finally, the decision variable T is evaluated by considering the following thresholds:

- Non-OSA case: Do not perform diagnostic studies: This status will be proposed when the decision variable has a value lower than two.
- Doubtful case: This status will be proposed when the decision variable equals two. The medical team should assess whether it is necessary to perform further examinations or suggest a new medical appointment after a period of time to reconsider the patient’s condition.
- Possible OSA case: Perform diagnostic studies.This status will be proposed when the decision variable is larger than, or equal to, three.

### 2.2. Implementation of the System

The intelligent decision support system described in Section 2.1.2 involves a series of stages from the collection of patient information and data processing through to the generation of alerts and decision making.

This section describes in detail the implementation of the intelligent system through a software artifact that verifies the recommendations of Hevner et al. [45,46] and, if considered, guarantees its future integration into a hospital information system.

Such implementation has been carried out using the MATLAB© programming environment (R2021b, MathWorks©, Natick, MA, USA), making use of the App Designer module [47] for the development of the graphical user interface, the Classification Learner [48] for training the machine-learning algorithms and the Fuzzy Logic toolbox [49] for the implementation of fuzzy logic inference systems. Furthermore, it was necessary to make an auxiliar use of Python’s (version 3.9.12) imbalanced-learn library [50] for synthetic data generation employing SMOTE-NC (Synthetic Minority Over-sampling Technique for Nominal and Continuous).

**Figure 3:**
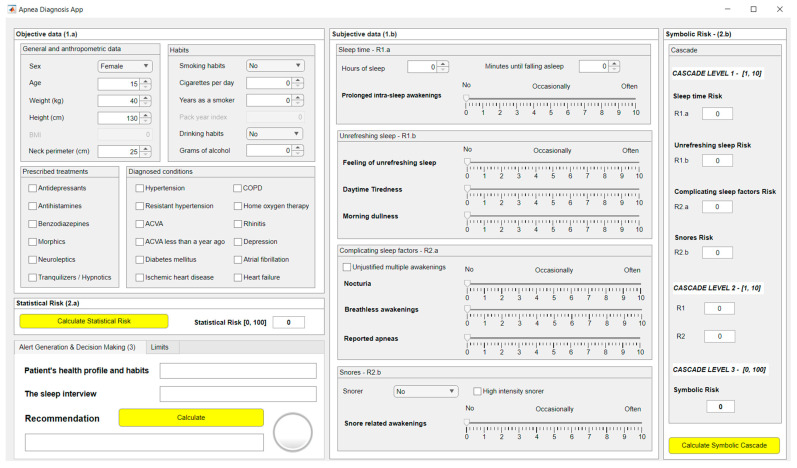
Screenshot of the application. (**1.a**) and (**1.b**) are related to the stage of collecting relevant patient information. (**2.a**) and (**2.b**) refer to the stage of data processing. (**3**) is related to generating alerts and recommendations.

Figure 3 shows a screenshot of the graphical interface of the developed software artifact. Block (1.a) is related to the compilation and preprocessing of objective patient information, while block (1.b) is related to the subjective information. Blocks (2.a) and (2.b) refer to the data processing, making it possible to observe the *Statistical Risk* and the *Symbolic Risk*, respectively. Block (3) allows the generation of alerts and visualization of the system recommendations.

#### 2.2.1. Data Acquisition

The data associated with each patient must be introduced into the application through the form shown in Figure 3. There are two areas in this, one for the introduction of objective data (1.a) and the other for the introduction of more subjective data (1.b). It is worth emphasizing the importance associated with the task of filling the forms, since errors or omissions in them could compromise the accuracy of the data, thus increasing the system’s uncertainty.

#### 2.2.2. Data Processing

After the patient’s data have been introduced into the application, the processing is performed by the intelligent system. As previously mentioned, two blocks that act concurrently [16,17,18,19,20,21,22] are used for this purpose. The first one is based on a machine-learning classification algorithm, while the second one is based on a series of cascaded expert systems.

The process used for the construction and definition of these blocks, as well as the determination of the associated risk metrics, are described below.

##### Classification Algorithm Based on Machine Learning

For the definition of the machine-learning classification algorithm, the dataset presented in Section 2.1.1 was used as a starting point; more specifically, the most objective data which is summarized in Table 1. As can be observed in the table, part of the data belongs to the nominal or ordinal categorical data types [51,52]. Because of this, an encoding has been made using *dummy encoding* [42], which means that for each variable, a number of auxiliary variables are created to replace it, equal to the total number of categories presented in the starting variable minus one. Moreover, it is also necessary to mention the numerical data, which were scaled from zero to one using Min–Max normalization (as shown in Equation (2)). This is done because, with the help of the medical team, it has been possible to delimit for each of the cases the maximum and minimum values between which the study variables will be encompassed.

(2) $$x\prime =\frac{x_{i}-\min\left(x\right)}{\max\left(x\right)-\min\left(x\right)}$$

After that, the distribution of the class to be predicted on the dataset is analyzed. As discussed in Section 2.1.1, considering an AHI threshold value of 15, it is observed that 2693 patients present a value equal to or higher than the threshold which are labeled as OSA cases. Meanwhile, 1707 patients present a lower value than the threshold and are labeled as non-OSA cases. Through the analysis of the dataset, a certain degree of imbalance is observed, which could affect the performance of the classifier. For this reason, a controlled data augmentation process is implemented as a usual approach in diagnostic environments, which tends to improve the results of binary classifiers [19,53]. A variation of the Synthetic Minority Over-Sampling Technique (SMOTE) was used for this purpose [53,54], oriented towards the processing of datasets in which numerical and categorical variables coexist, in this case the SMOTE-NC technique (Synthetic Minority Over-sampling Technique for Nominal and Continuous). Data of both classes have been generated with a strategy where the number of neighbors k = 5 was chosen until there were 4000 elements of each class. This provides a coherent training data set that can be used for the training of machine-learning-based classification algorithms, which makes it possible to classify new patients. To this end, and in order to evaluate the different available possibilities, a series of tests have been carried out using the MATLAB© Classification Learner app. This allows the training and analysis of multiple algorithms in a massive way, establishing a k-fold cross-validation strategy [55] with k = 5.

**Figure 4:**
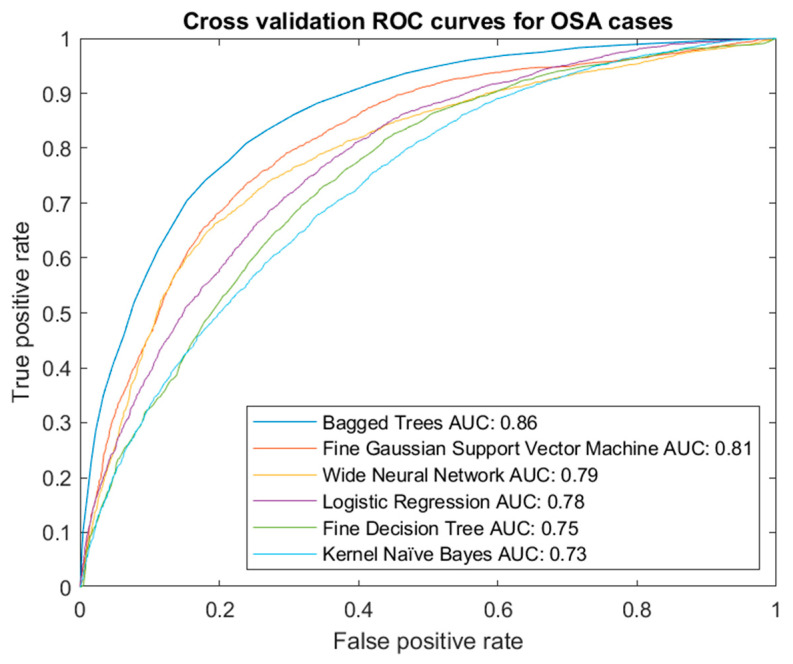
Cross-validation ROC curve for OSA cases.

Different types of models were tested, including decision trees, logistic regression, naïve Bayes, support vector machines, ensembles (in this case, Bagged Trees) or artificial neural networks, among others. Figure 4 shows a summary graph of the ROC curves from the best models for the OSA cases.

**Figure 5:**
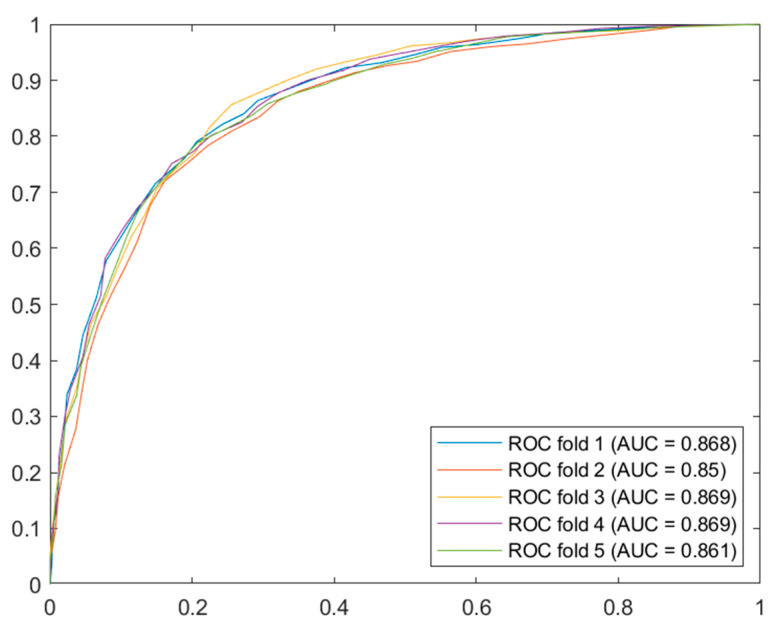
ROC curves and AUC values for each of the folds of Bagged Trees for OSA cases.

Once the analysis of the results was performed, by interpreting the validation ROC curves, the Bagged Trees algorithm stands out. It should be noted that using one algorithm or another does not constrain the system in any way and that, in the future, if it is found that other algorithms give better results, they could be replaced without causing an essential change in the system. In any case, the stability of the chosen model was verified by simulating the calculation of the ROC curve and the AUC value in each fold, with minimal differences between them, as can be seen in Figure 5.

**Figure 6:**
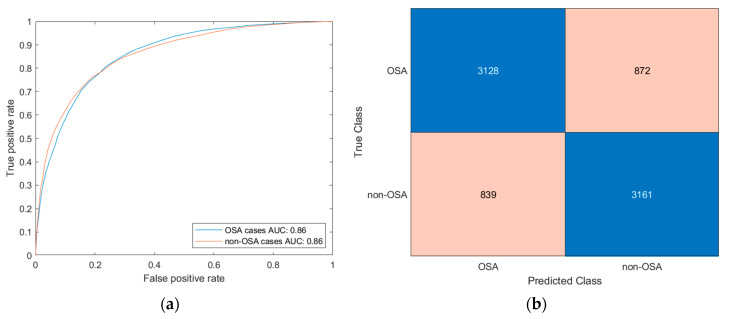
(**a**) Validation ROC curves for the Bagged Trees model. (**b**) Confusion matrix for the Bagged Trees model.

Figure 6a shows the ROC validation curve of the Bagged Trees algorithm for both classes, which show an AUC value close to 0.90. Figure 6b shows the model’s confusion matrix.

At this point, the next step consists in feeding data from a new patient into the classifier and obtaining a risk indicator, the *Statistical Risk*. This output is associated with the percentage risk of suffering from OSA for an AHI value greater than, or equal to, the determined threshold level, 15 in this case. This risk is scaled from 0 to 100, with 0 being the minimum percentage of having an AHI greater than or equal to the threshold, and 100 being the highest one.

##### Cascade of Expert Systems

Concurrent with the machine-learning module [16,17,18,19,20,21,22], in which the *Statistical Risk* was determined, in this module, the *Symbolic Risk* is calculated. For this purpose, a cascade of expert systems (introduced in Section 2.1.2) is deployed using Mamdani-type fuzzy inference system [23,24,25,26]. As shown in Figure 2, the cascade has three levels, which are detailed below:

- First level: At the upper level of the cascade, the processing of the four groups of information previously introduced in Stage 2.b of Section 2.1.2 is carried out (‘sleep time’ group, ‘unrefreshing sleep’ group, ‘complicating sleep factors’ group and ‘snores’ group). For this purpose, four expert systems are used to obtain a risk indicator (R1.a, R1.b, R2.a and R2.b, respectively) as an output after the defuzzification process. These indicators determine the risk level associated with suffering from OSA related to each group of data.
- Second level: At the second level of the cascade, the data from the first level is processed using two expert systems with the aim of aggregating their outputs. This is so because of the decision to group the risks obtained in the first level of the cascade into couples (R1.a and R1.b, R2.a and R2.b) according to the degree of affinity between the starting data. As a result, two risk indicators related to the groups of data linked to each indicator (R1 and R2, respectively) which show the risk associated with suffering from OSA are determined at the output of the expert systems after the defuzzification process.
- Third level: At the third level of the cascade, the data from the second level of the cascade (R1 and R2) are processed using a single expert system. At its output, after the defuzzification process, a risk indicator is obtained, the *Symbolic Risk*, which indicates the risk level associated with the patient suffering from OSA according to the subjective input data.

The use of the cascade of expert systems makes it possible to aggregate the information of the different levels in a progressive way. The information related to the different criteria contemplated, understood as the different groups of data involved in the evaluation of the risk of suffering from OSA, can be incorporated. In addition, the use of a cascade-type structure facilitates the determination of the rules of each inference system. As the number of antecedents in the expert systems is smaller, greater precision is obtained in the elaboration of the rules.

**Figure 7:**
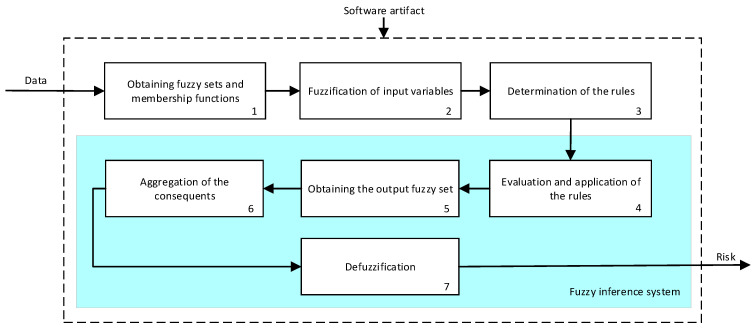
Functional diagram of the inference system.

As already mentioned, all the expert systems from the cascade are based on the Mamdani-type fuzzy inference system [23,24,25,26]. Figure 7 shows the operation’s flow diagram for this type of inference system, which is described in detail below.

First of all, the membership functions are determined for each of the variables. This make it possible to establish the degree of membership associated with a new value of a variable, with a value between zero (indicating non membership) and one (indicating absolute membership). As already mentioned, in the expert systems of the first level, the input variables are those described in Table 2, while in the second and third levels of the cascade, the inputs are the risks obtained after the defuzzification process of the expert systems of the immediately preceding level. With regards to the expert systems’ outputs, in this case, different risk indicators associated with the initial data will be obtained. The choice of one type of membership function or another will depend on the characteristics of the variable to be represented. Following Ross’s recommendation [26], normal, convex and symmetrical membership functions will be used, choosing, in this case, between triangular and trapezoidal functions [19]. After defining the membership functions, the next step is the fuzzification of the new input values to determine a series of membership degrees associated with each of them. Once this is done, in the third stage, the knowledge base of the system is established, wich is composed of a collection of declarative rules determined by the medical team. These rules are of the type ‘IF … AND … THEN …’, through which it is possible to represent the knowledge of the experts by combining the different input variables and relating them to the consequents. The fourth stage then evaluates the antecedents of the rules of the Mamdani system. As in the case of this study, when different membership functions are connected through the ‘AND’ operator, the lowest of the membership degrees associated with each of them will be obtained. After evaluating the antecedents, in the fifth stage, the next step involves obtaining the consequents by applying an implication method, in this case, the ‘minumum’, which truncates the membership function of the consequents of each rule. These truncated consequents are subsequently aggregated in the sixth stage by applying a disjunctive approach [26] based on the use of the ‘maximum’ operator, so as to achieve a graphical output equivalent to the superposition of the previously obtained consequents. This is subsequently defuzzified in the last stage by applying the centroid method [26] to determine a numerical value associated with the risk indicator at each of the risk levels. Nonetheless, the system contemplates that the variables and membership functions can be redefined based on the experience acquired during the use of the application.

Table 3, Table 4, Table 5 and Table 6 below summarize the initial configuration of the expert systems of the first level of the cascade, which are used for the calculation of risks R1.a, R1.b, R2.a and R2.b, respectively.

In the same way, Table 7 and Table 8 show the initial configuration of the expert systems of the second level of the cascade, which are used for the calculation of risks R1 and R2.

Finally, Table 9 shows the initial configuration of the expert system of the third level of the cascade, which is used for the calculation of the *Symbolic Risk*.

After the *Symbolic Risk* is obtained, it is rescaled in the range [0, 100] so that it can be compared to the *Statistical Risk* on the same scale. A higher value of the *Symbolic Risk* indicates a higher risk level of suffering from OSA.

#### 2.2.3. Generation of Alerts and Decision Making

In the case of a new patient, and after determining both the *Statistical Risk* and *Symbolic Risk* indicators, the patient’s condition is determined, and a recommendation is proposed. As mentioned, the risk indicators will be first interpreted individually based on a series of threshold values that allow a risk level associated with each of the indicators to be established. The second column of Table 10 shows a summary of the different possible cases and their correspondence to each different level.

Regarding both the *Statistical Risk* and the *Symbolic Risk*, the value of Limit 1 is proposed to be 45, while the value of Limit 2 has been set at 65 for *Symbolic Risk*, and 60 for *Statistical Risk*. Nevertheless, these values may be reviewed and modified depending on the results observed during the clinical validation of the system.

Subsequently, the risk levels associated with each of the risk indicators will be available, and their joint evaluation will be carried out in order to establish a recommendation. Before that, a score will be assigned to each of the levels as can be seen in the third column of Table 10. Once this has been done, and by adding the score associated with the risk levels, a decision variable T will be determined as shown in Equation (3). By default, both scores are given equal weight ($W_{ST}=W_{SY}=1$), but it might be possible to increase the effect of the terms used to calculate it by using weighting coefficients ($W_{ST}\;and\;W_{SY}\in \left[0,2\right]$; and $W_{ST}=2-W_{SY}$).

(3) $$T\;=W_{ST}\cdot Statistical_{score}+W_{SY}\cdot Symbolic_{score}$$

This decision variable T could be considered as part of the usefulness analysis, representing a normative tool as opposed to the descriptive measure offered by the calculated risk values. Its objective is therefore not to predict but to assist in decision making by establishing a relationship between the calculation of risks and the preferential recommendation associated with the value of T and linked by Equation (1), which, in this setting and to this end, could be considered as an utility function [56,57].

Finally, the value of the decision variable T is evaluated. Table 11 presents a summary of the different recommendations proposed according to the value of the decision variable T. Emphasis should be placed on the fact that the threshold values for the variable T can be reviewed and modified according to the results obtained.

To summarize, Table 12 shows the whole process of generating alerts and decision making from the individual evaluation of each of the indicators determining the risk levels to their joint evaluation in determining the T variable and establishing the conclusions and recommendations. In this table, color codes have been used for the generation of alerts once the T variable has been evaluated. The green color is related to a non-OSA case and orange to a doubtful case, while red refers to a potential OSA case.

## 3. Results

This section presents a clinical case study of the application of the intelligent decision support system proposed in this paper. The aim is to give an example of its performance and potential use in the clinical field. It is important to clarify that the intention is neither to validate the system nor to compare it with other alternatives existing in the current body of knowledge.

Furthermore, prior to the presentation of the case study, it should be pointed out that the patient data analyzed in this section was not present in the dataset used for the definition and configuration of the system.

### 3.1. Compilation of the Patient’s Information

Table 13 shows the objective data of the patient to be analyzed, which is related to Stage 1.a of the proposed system. Table 14 presents the subjective data related to Stage 1.b of the proposed system. It is necessary to point out that this patient underwent sleep studies and presented an AHI value of 11.90. This value will be later used to evaluate the conclusions and recommendations generated by the intelligent system.

Once the data were submitted, they were introduced into the application to be processed by the intelligent clinical decision support system.

### 3.2. Data Processing

**Figure 8:**
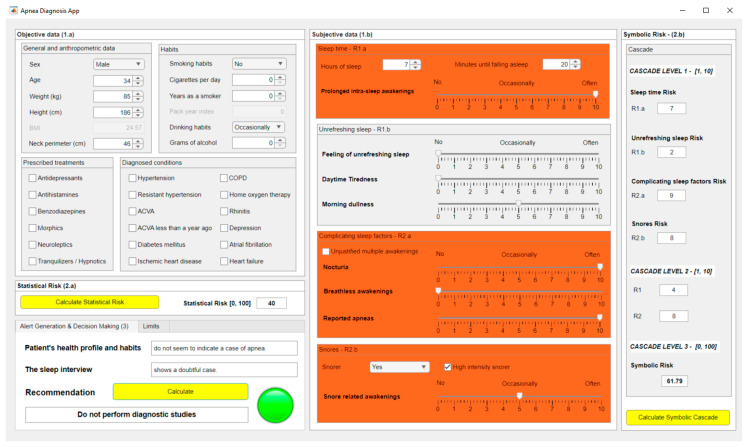
Screenshot of the application.

Subsequently, the two risk indicators previously defined in Section 2, *Statistical Risk* and *Symbolic Risk*, were determined. Figure 8 shows a screenshot of the application in which it is possible to observe the resulting risk values. In the case of *Statistical Risk* a percentage value of 40 was obtained, while in the case of the *Symbolic Risk* the respective value was 61.79, both of them expressed on a scale from 0 to 100.

The *Symbolic Risk* should be analyzed in more detail since, as mentioned in Section 2, it is the final value obtained from the cascade of expert systems. Analyzing the systems of the first level of the cascade, risk values of 7, 2, 9 and 8 were respectively obtained for risk indicators R1.a, R1.b, R2.a and R2.b, as shown in Figure 8.

Next, in the second level of the cascade, risk values of 4 and 8 were obtained for risk indicators R1 and R2, respectively. These risks, R1 and R2, were used for the calculation of the *Symbolic Risk* at the last level of the cascade, resulting in a preliminary value of 6.179, which, after being scaled from 0 to 100, presented a value of 61.79.

### 3.3. Generation of Alerts and Decision Making

After entering the patient’s data into the application and calculating the risk indicators associated with them, i.e., the *Statistical Risk* and the *Symbolic Risk*, that last stage was followed by their analysis and evaluation.

Both risks were first evaluated against three levels, each of them defined by two limit values. In this case, the limits are shown in Table 15. The value of Limit 1 was set at 45 in both cases, while the value of Limit 2 was set at 65 for *Symbolic Risk*, and 60 for *Statistical Risk*. A summary of the thresholds associated with the different levels, as well as their respective interpretations, is shown in Table 15.

The *Statistical Risk* showed a value of 40, which is lower than Limit 1, so this indicator is at the first level corresponding to a patient who does not suffer from OSA. On the other hand, the *Symbolic Risk* presented a value of 61.79, higher than Limit 1 and lower than Limit 2, so it is at the second level and associated with a doubtful case.

Once the individual interpretation of the risk indicators has been carried out, which is done automatically in the application based on the established thresholds, the next step is their joint interpretation. To understand the procedure, it may be helpful to retrieve Table 12, adapting it to this case as shown in Table 16, which allows to determine the recommendation of the system through the color code in that table (green for non-OSA, orange for a doubtful case, and red for a potential OSA case).

As the interpretation in Table 16 shows, an OSA case is not considered, so it is suggested not to perform further diagnostic studies. This final interpretation is also performed automatically by the system, as can be seen in Figure 8.

In any case, it is interesting to make a brief analysis of the results obtained. After the interpretation of the *Statistical Risk,* it is apparent that this is not a case that fits the usual pattern of an OSA patient, given that the risk value is relatively low.

Nevertheless, the patient presents some significant risk values in the cascade, such as R2.a and R2.b, associated with the ‘sleep complicating factors’ and ‘snores’ data groups, respectively. This may be due either to the patient not telling the truth or exaggerating their symptoms. Thus, after the joint assessment of the different levels of the cascade, the value of the *Symbolic Risk* obtained is average, which indicates that this case would be a doubtful one.

Following the joint assessment of the indicators, it was determined that the patient did not suffer from OSA, which is feasible given that the patient had an AHI value close to 10, commonly found in mild cases. Furthermore, it should be noted that the machine-learning classification algorithm was trained using a dataset with an AHI threshold value of 15. This was set so as to discriminate mild cases from moderate–severe ones.

### 3.4. Expansion of the Results

**Figure 9:**
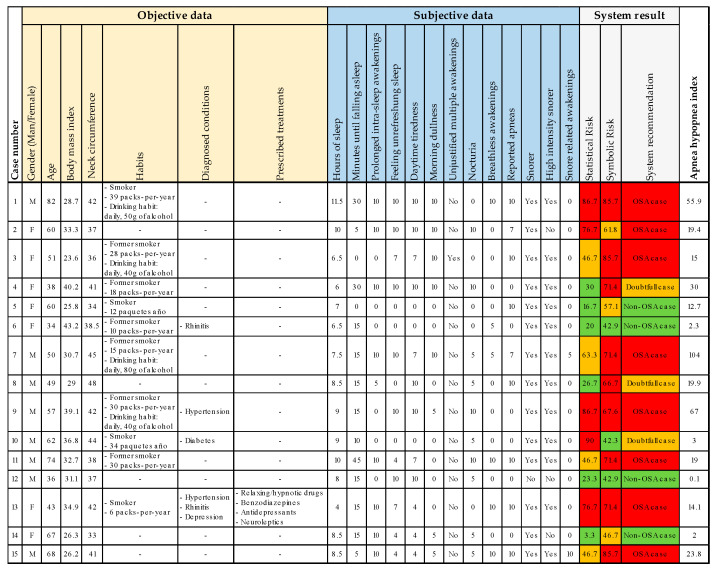
Expansion of the case study. In the case of the subjective data, the values were indicated using the scales (generally, 0 means ‘No’; 5 means occasionally; and 10 means ‘often’). The background colors in the ‘System result’ section relate to the three levels of risk (green = L1; orange = L2; red = L3) and the interpretation of the system recommendation (green = non-OSA case; orange = doubtful case; red = OSA case).

To extend the case study, fifteen new cases are presented below in Figure 9, following a similar process to that one previously described in Section 3. All cases analyzed were referred from primary care or other specialist areas as suspected OSA cases. The figure shows the system input data, as well as the results obtained and the AHI level. The results are color-coded for both the risk analysis (green = L1; orange = L2; red = L3) and the interpretation of the system’s recommendation (green = non-OSA case; orange = doubtful case; red = OSA case).

## 4. Discussion

Currently, OSA has a high incidence worldwide and involves a significant detriment to the health of those who suffer from it, notably increasing the demand for related medical consultations and diagnostic studies. These studies present a high instrumental complexity, it being necessary to use large numbers of sensors as well as to be supervised by specialized professionals, in addition to the requirement for a subsequent manual analysis of the results. As a consequence of the increasing demand for these types of studies, as well as the particularities inherent to these types of tests, considerable delays are frequent in their use. This entails a severe hazard to the health of the patients, together with the economic impact associated with the performance, in many cases, of tests that are not necessary. In this regard, and considering the important advances in the field of artificial intelligence, numerous and diverse approaches have been proposed in recent years for OSA diagnosis, generally based on the use of single (statistical in most cases) inference engines.

The diagnosis of OSA is a multivariate problem in which the aim is to assess whether a patient suffers from this clinical condition on the basis of a series of variables. Another purpose may be to determine potential cause–effect relationships that exist between the different variables involved, for which it is common to use dependence, interdependence or structural approaches [58]. Nevertheless, it is also feasible to deal with this type of problem by jointly employing inferential models of a heterogeneous nature [59,60,61,62,63], both statistical and symbolic, with the common objective of representing the same reality. In this case, the diagnostic process that allows for the discernment between a patient who suffers from OSA and one who does not. For this purpose, in the case of the statistical inferential approaches represented in this work through the use of the Bagged Trees algorithm, which is applied to determine the *Statistical Risk*, it is essential to have a representative dataset available to build the model. Meanwhile, in the case of the symbolic inferential approach particularized in this work through the use of a set of expert systems based on fuzzy logic inferential engines, through which it is possible to determine the *Symbolic Risk*, it is necessary to define the knowledge base through a series of rules. In both cases, the definition of each of the systems used contemplates a large number of variables that are not free of uncertainty. This is where the proposed intelligent system becomes essential. Beyond its undeniable applicability and potential usefulness in clinical settings, the capabilities of the different elements used for the determination of each of the system’s risks are evaluated, taking into account their ability to represent knowledge and manage uncertainty:

- Determination of *Statistical Risk*: As previously mentioned, a machine-learning classification algorithm, more specifically, a Bagged Trees algorithm, is used to determine the *Statistical Risk*, built on the basis of an initial dataset that has been encoded, normalized and balanced using SMOTE-NC. All this process prior to the construction of the model has been carried out with the aim of ensuring that the initial dataset used for the construction of the model is coherent and adequate. An attempt has been made to achieve sufficient representativeness regarding the possible casuistry as well as to aim for normality in the data distributions in order to guarantee the subsequent obtaining of robust and reliable classifiers. As mentioned before, in this work, a Bagged Trees algorithm was chosen; however, the use of one algorithm or another is not relevant because any other machine-learning approach could provide plausible results. In any case, it should be noted that for this to be true, the datasets used in the model training and validation processes must have been obtained under similar circumstances, with common diagnostic criteria. The same circumstances should apply when it is desired to analyze data from new patients. In relation to the treatment of uncertainty, in this case, it is achieved using a purely probabilistic approach.
- Determination of the *Symbolic Risk*: Concurrent with the calculation of the *Statistical Risk*, the *Symbolic Risk* is determined using a series of expert systems, which are perhaps the most representative models for symbolic reasoning in the field of artificial intelligence and which allow for the diversifying and formalizing of the experts’ knowledge. In this case, the formalization of knowledge has been achieved through the definition of an architecture of expert systems arranged in cascade. The diversification of information is possible through the definition of a series of declarative rules in each of the expert systems that model the knowledge of events that have occurred in similar circumstances. Thus, there is a clear and undeniable dependence between the way in which the expert system performs its reasoning and who defines its knowledge base; this implies assuming a certain degree of doubt and error in the process, and therefore, the presence of uncertainty in the generation of the rules. The formalization of knowledge is an inherent characteristic of expert systems, and it is possible to do so in this case through the definition of a cascade-based architecture. This allows the gradual integration of the consequents of the previous levels, all of which are considered technical variables representing the risk of suffering from OSA. These consequents are treated, in turn, as qualitative variables when acting as antecedents of the expert systems in the next level. As discussed in the work by Casal-Guisande et al. [19], the distinction that the same variable may be treated as antecedent or consequent of a rule makes a clear difference in the very fuzziness of the variable, which is related to the uncertainty associated with its numerical representation. In addition, the cascaded expert system architecture allows for simpler logical constructs, through which it is possible to represent knowledge. That also results in better control and in the progressive reduction in uncertainty throughout the different stages of the cascade. It is because of all those reasons that the intelligent system, in its symbolic aspect, has capabilities to manage uncertainty.

Beyond those issues related to the architecture of the proposed intelligent system, as well as its ability to manage uncertainty, it is necessary to point out those aspects that are most beneficial from a diagnostic and practitioner’s viewpoint. Once the risks have been obtained, their analysis and interpretation provide the medical team with a metric for the hazard level derived from the patient’s risk of suffering from OSA. Such an assessment is based both on objective data related to the patient’s history and on subjective data related to the symptoms reported by the patient. This information is remarkably valuable due to the fact that it facilitates the assessment of patients suspected of suffering from OSA. Furthermore, it could be very useful for those first medical consultations by which the patient comes to primary care, where a general practitioner could suspect a potential OSA case. This could help them to choose which cases should be referred to further specialized studies, thus reducing the overall number of referred patients, as well as focusing on those who are actually in need. This is possible thanks to the system’s ability to formalize and diversify knowledge, guiding the physician, standardizing the diagnostic process and facilitating the interpretation of data. Likewise, the system also has a great potential for its use in specialized units, being of particular interest in those cases in which specialists are faced with doubtful cases. The system enables them to discriminate between those patients who may require further diagnostic studies to confirm a potential OSA case, and those who apparently do not have that disease. In this way, the demand for sleep studies may be reduced, thus speeding up the performance of these studies while reducing waiting lists at the same time.

On a general note, and in line with what has already been mentioned, it should be pointed out that the tool presented in this article constitutes a great novelty in the field of study. Existing approaches in the current body of knowledge generally make use of single inference models, statistical in most cases, being clearly dependent on the availability of coherent and representative population healthcare databases. This could be a severe handicap for some diseases, as might be the case for OSA. Furthermore, the relevant impact of using these types of systems in the management of hospital resources, as well as on their associated cost savings, should be highlighted once again.

### Relevance of the Proposal in the Field of Study

After the detailed discussion and analysis of the relevance and technical soundness of the essential components of the proposed intelligent system, a comparison with other existing state-of-the-art approaches is presented in Table 17, taking into account a set of common criteria used in the field of intelligent systems [19,20,21,64]:

- Efficiency: this refers to the reliability of the results obtained, understood as the capabilities of the intelligent system to deal with uncertainty.
- Scalability: this refers to the versatility and capabilities of the system to replace or remove the current inferential engines.
- Reasoning: this is related to the system’s ability to perform symbolic reasoning.
- Learning: this refers to the system’s capability to incorporate learning approaches, which is common in the field of machine learning.

In general, when analyzing the main reviews about screening approaches in the field of OSA [9,65,66,67], and considering the works analyzed in Table 17, there is a clear trend towards the use and development of decision support systems being applied to the diagnosis of OSA that are based on the use of machine-learning techniques. Nevertheless, there are some works that use symbolic inference approaches, such as the one proposed by C. Zoroglu and S. Turkeli [41], or the other one proposed by J. M. Matthews et al. [42], or others that, although mainly using learning-based approaches, aim to integrate the benefits associated with symbolic inference [21], allowing a formalization of knowledge and a better management and handling of the uncertainty present in the diagnostic process. For all these reasons, the proposed system represents a clear novelty in the OSA diagnostic field, extending the capabilities of the systems usually present in this area.

## 5. Conclusions

In this article a novel intelligent decision support system applied to the diagnosis of OSA has been presented, which allows for the optimizing of the diagnostic process of potential OSA cases. To this end, statistical and symbolic inferential approaches were used together, making it possible to determine two risk indicators, the *Statistical Risk* and the *Symbolic Risk*, both of them associated with suffering from OSA.

The proposed intelligent system was exemplified in a case study as a proof of concept, which provided an introduction to the tool, demonstrating the use of the system, and highlighting both its simplicity of use and its great applicability in the field of study. Despite the aforementioned claims and the encouraging results obtained, it is worth mentioning that the proposed system is still in its early stages of development, and it is still in need of further clinical validation.

In time to come, it will be necessary to carry out tests in clinical settings to validate the results obtained and to adjust the system for its intensive use in hospital environments, and comparing its outcomes with the clinical guidelines in the field of study. Thus, it will be possible to determine its full diagnostic capabilities and the economic impact associated with its use. In addition, and from the point of view of the system’s architecture, it will still be necessary to explore new options to improve the final process of joining the risks obtained, as well as to optimize the formalization of the knowledge of the symbolic models.

## Figures and Tables

**Table 1:** Summary of the objective data.

Subgroup	Data	Data Type	Commentary
General and anthropometric data	Sex	Categorical	Male/Female
	Age	Numerical	-
	Weight	Numerical	Not provided to the algorithm but used to determine the body mass index (BMI)
	Height	Numerical	Not provided to the algorithm but used to determine the body mass index (BMI)
	Body mass index (BMI)	Numerical	Data derived from height and weight
	Neck circumference	Numerical	-
**Subgroup**	**Data**	**Data Type**	**Commentary**
Habits	Smoker	Categorical	Yes/No/No longer
	Cigarettes per day	Numerical	Not provided to the algorithm but used to determine the pack year index
	Years as a smoker	Numerical	Not provided to the algorithm but used to determine the pack year index
	Pack-year index	Numerical	Data derived from cigarettes per day and years smoking
	Drinking habits	Categorical	No/Daily/Occasionally
	Grams of alcohol	Numerical	-
**Subgroup**	**Commentary**		
Diagnosed conditions	All the comorbidities listed in Section 2.1.1 are included. Each of these fields is considered as categorical or binary, that is, either the pathology is suffered or not		
**Subgroup**	**Commentary**		
Prescribed treatments	All the drugs listed in Section 2.1.1 are included. Each of these fields is considered as categorical or binary, that is, prescribed treatments are or are not provided		

**Table 2:** Summary of the subjective data.

Subgroup	Data	Data Type	Commentary
Sleep time	Hours of sleep	Numerical	-
	Minutes until falling asleep	Numerical	-
	Prolonged intra-sleep awakenings	Categorical	No/Occasionally/Often
**Subgroup**	**Data**	**Data Type**	**Commentary**
Unrefreshing sleep	Feeling of unrefreshing sleep	Categorical	No/Occasionally/Often
	Daytime tiredness	Categorical	No/Occasionally/Often
	Morning dullness	Categorical	No/Occasionally/Often
**Subgroup**	**Data**	**Data Type**	**Commentary**
Complicating sleep factors	Unjustified multiple awakenings	Categorical	Yes/No
	Nocturia	Categorical	No/Occasionally/Often
	Breathless awakenings	Categorical	No/Occasionally/Often
	Reported apneas	Categorical	No/Occasionally/Often
Snores	Snorer	Categorical	No/In supine position only/Yes
	High intensity snorer	Categorical	Yes/No
	Snoring-related awakenings	Categorical	No/Occasionally/Often

**Table 3:** Initial configuration of the inference system responsible for processing the ‘sleep time’ data group.

Inference System Associated with the ‘Sleep Time’ Data Group.			
Input Data	Range	Output Risk	Range
Hours of sleep	0–14 h	R1.a	0–10
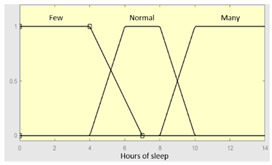		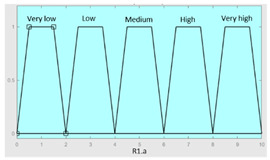	
Minutes until falling asleep	0–240 min	**Initial configuration**	
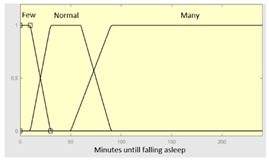		Fuzzy structure: Mamdani-type Membership function type: trapezoidal Defuzzification method: centroid [26] Implication method: MIN. Aggregation method: MAX. Number of fuzzy rules: 46	
Prolonged intra-sleep awakenings	0–10	**Subset as an example of the 46 fuzzy rules**	
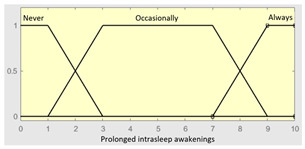		1. IF (Hours_of_sleep is Few) AND (Minutes_until_falling_asleep is Few) AND (Prolonged_intra-sleep_awakenings is Never) THEN (R1.a is Low). 2. IF (Hours_of_Sleep is Few) AND (Minutes_until_falling_asleep is Few) AND (Prolonged_intra-sleep_awakenings is Never) THEN (R1.a is Medium).	
**Graphical example of fuzzy rules 1 and 2**			
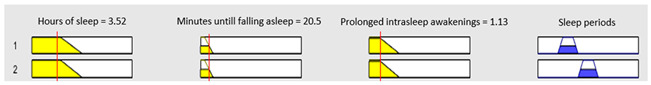			

**Table 4:** Initial configuration of the inference system responsible for processing the ‘unrefreshing sleep’ data group.

Inference System Associated with the ‘Unrefreshing Sleep’ Data Group			
Input Data	Range	Output Risk	Range
Feeling of unrefreshing sleep	0–10	R1.b	0–10
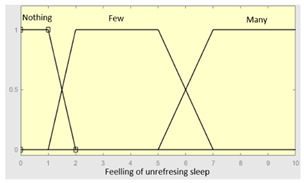		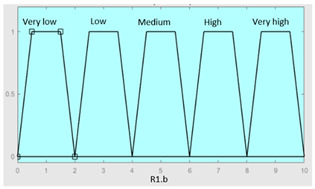	
Daytime tiredness	0–10	**Initial configuration**	
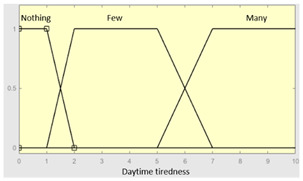		Fuzzy structure: Mamdani-type Membership function type: trapezoidal Defuzzification method: centroid [26] Implication method: MIN. Aggregation method: MAX. Number of fuzzy rules: 45	
Morning dullness	0–10	**Subset as an example of the 45 fuzzy rules**	
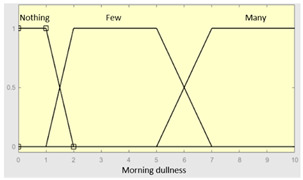		1. IF (Feeling_of_unrefreshing_sleep is Nothing) AND (Daytime_tiredness is Nothing) AND (Morning_dullness is Nothing) THEN (R1.b is Very_low). 2. IF (Feeling_of_unrefreshing_sleep is Nothing) AND (Daytime_tiredness is Nothing) AND (Morning_dullness is Few) THEN (R1.b is Very_low).	
**Graphical example of fuzzy rules 1 and 2**			
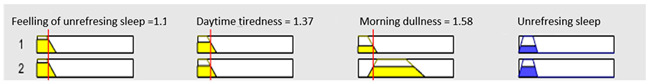			

**Table 5:** Initial configuration of the inference system responsible for processing the ‘complicating sleep factors’ data group.

Inference System Associated with the ‘Complicating Sleep Factors’ Data Group			
Input Data	Range	Output Risk	Range
Unjustified multiple awakenings	0–1	R2.a	0–10
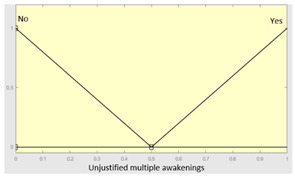		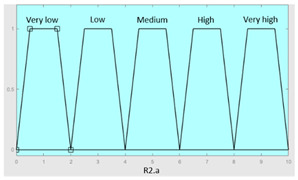	
Nocturia	0–10	**Initial configuration**	
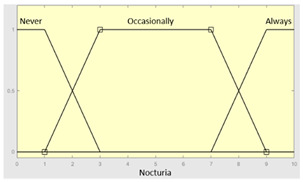		Fuzzy structure: Mamdani-type Membership function type: trapezoidal Defuzzification method: centroid [26] Implication method: MIN. Aggregation method: MAX. Number of fuzzy rules: 78	
Breathless awakenings	0–10		
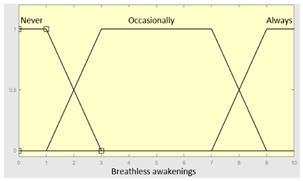			
Reported apneas	0–10	**Subset as an example of the 78 fuzzy rules**	
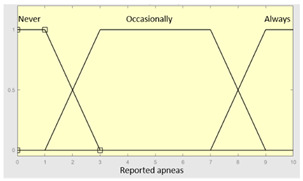		1. IF (Reported_apneas is Always) THEN (R2.a is Very_high). 2. IF (Unjustified_multiple_awakenings is No) AND (Nocturia is Never) AND (Beathless_awakenings is Never) AND (Reported_apneas is Never) THEN (R2.a is Very_low).	
**Graphical example of fuzzy rule 1**			
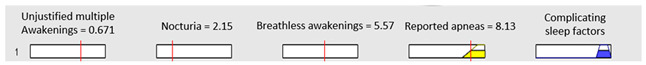			

**Table 6:** Initial configuration of the inference system responsible for processing the ‘snores’ data group.

Inference System Associated with the ‘Snores’ Data Group			
Input Data	Range	Output Risk	Range
Snorer	0–2	R2.b	0–10
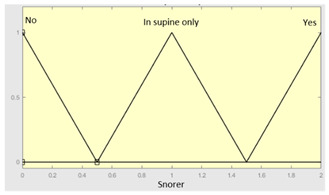		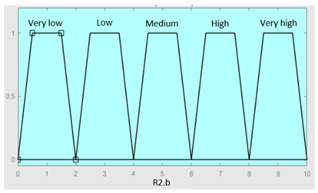	
High intensity snorer	0–1	**Initial configuration**	
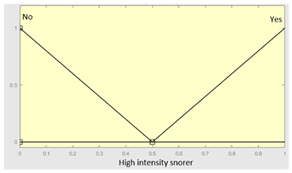		Fuzzy structure: Mamdani-type Membership function type: trapezoidal Defuzzification method: centroid [26] Implication method: MIN. Aggregation method: MAX. Number of fuzzy rules: 30	
Snore-related awakenings	0–10	**Subset as an example of the 30 fuzzy rules**	
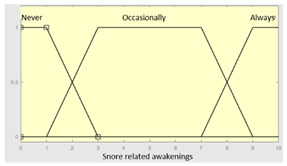		1. IF (Snorer is No) THEN (R2.b is Very_low). 2. IF (Snorer is In_dorsal_position_only) AND (High_intensity_snorer is No) AND (Snore_related_awakenings is Never) THEN (R2.b is Very_low).	
**Graphical example of fuzzy rule 1**			
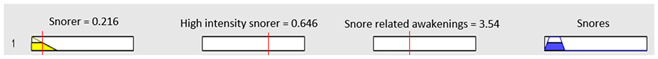			

**Table 7:** Initial configuration of the inference system responsible for processing risks R1.a and R1.b.

Inference System for the Processing of Risks R1.a and R1.b			
Input Data	Range	Output Risk	Range
R1.a	0–10	R1	0–10
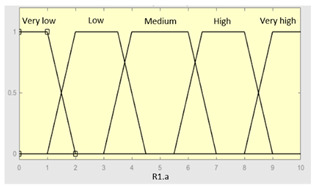		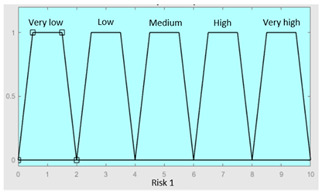	
R1.b	0–10	**Initial configuration**	
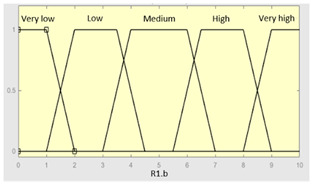		Fuzzy structure: Mamdani-type Membership function type: trapezoidal Defuzzification method: centroid [26] Implication method: MIN. Aggregation method: MAX. Number of fuzzy rules: 49	
**Subset as an example of the 49 fuzzy rules**			
IF (R1.a is Very_low) AND (R1.b is Very_low) THEN (R1 is Very_low). IF (R1.a is Very_low) AND (R1.b is Low) THEN (R1 is Very_low). IF (R1.a is Very_low) AND (R1.b is Low) THEN (R1 is Low).			
**Graphical example of fuzzy rules 1, 2 and 3**			
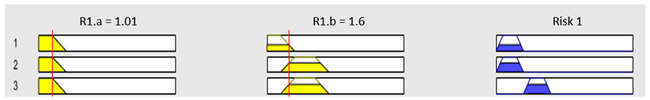			

**Table 8:** Initial configuration of the inference system responsible for processing risks R2.a and R2.b.

Inference System for the Processing of Risks R2.a and R2.b			
Input Data	Range	Ouput Risk	Range
R2.a	0–10	R2	0–10
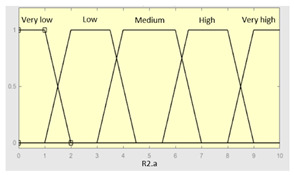		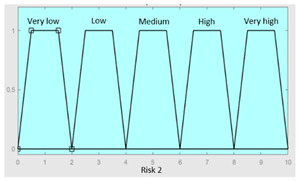	
R2.b	0–10	**Initial configuration**	
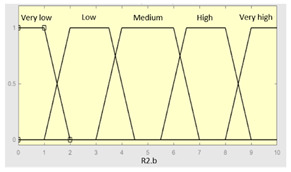		Fuzzy structure: Mamdani-type Membership function type: trapezoidal Defuzzification method: centroid [26] Implication method: MIN. Aggregation method: MAX. Number of fuzzy rules: 57	
**Subset as an example of the 57 fuzzy rules**			
IF (R2.a is Very_low) AND (R2.b is Very_low) THEN (R2 is Very_low). IF (R2.a is Low) AND (R2.b is Very_low) THEN (R2 is Very_low). IF (R2.a is Low) AND (R2.b is Very_low) THEN (R2 is Low).			
**Graphical example of fuzzy rules 1, 2 and 3**			
** 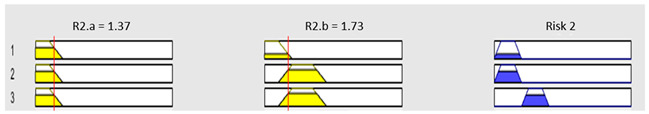 **			

**Table 9:** Initial configuration of the inference system responsible for processing risks R1 and R2.

Inference System for the Processing of Risks R1 and R2			
Input Data	Range	Output Risk	Range
R1	0–10	Symbolic Risk	0–10
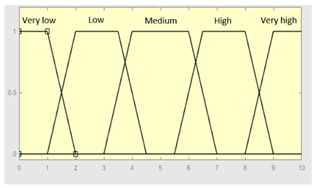		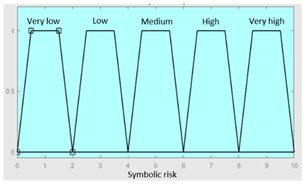	
R2	0–10	**Initial configuration**	
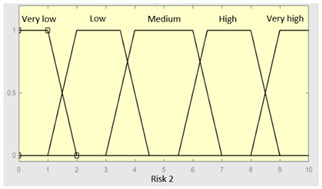		Fuzzy structure: Mamdani-type Membership function type: trapezoidal Defuzzification method: centroid [26] Implication method: MIN. Aggregation method: MAX. Number of fuzzy rules: 57	
**Subset as an example of the 57 fuzzy rules**			
IF (R1 is Very_low) AND (R2 is Very_low) THEN (Symbolic_risk is Very_low). IF (R1 is Very_low) AND (R2 is Low) THEN (Symbolic_risk is Very_low). IF (R1 is Very_low) AND (R2 is Low) THEN (Symbolic_risk is Low).			
**Surface**			
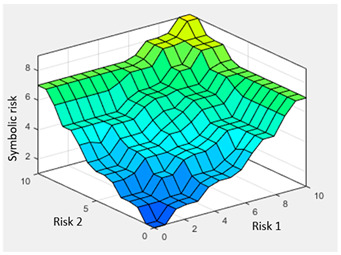			

**Table 10:** Risk assessment thresholds and scores.

Level	Case	Score
Level 1	If Risk < Limit 1 (L1)	0
Level 2	Limit 1 (L1) ≤ Risk < Limit 2 (L2)	1
Level 3	If Risk ≥ Limit 2 (L2)	2

**Table 11:** Summary of recommendations.

Case	Recommendation
T < 2	Non-OSA case—do not perform diagnostic studies
T = 2	Doubtful case—medical team should assess whether further tests or a new medical evaluation after a period of time is necessary to reconsider the patient’s condition.
T ≥ 3	Possible OSA case—perform diagnostic studies

**Table 12:** Graphical representation of the assessment process. The colours refer to the recommendations suggested by the system. The green colour indicates a non-OSA case; the orange colour indicates a doubtful case; the red colour indicates a possible OSA case.

	**Statistical Risk**	Risk < L1	L1 ≤ Risk < L2	Risk ≥ L2
	**Symbolic Risk**	Risk < L1	L1 ≤ Risk < L2	Risk ≥ L2
	Level & Score	Level 1 (0)	Level 2 (1)	Level 3 (2)
	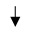			
	**Symbolic Risk**			
**Statistical** **Risk**	Level & Score	Level 1 (0)	Level 2 (1)	Level 3 (2)
	Level 1 (0)	0 + 0	0 + 1	0 + 2
	Level 2 (1)	1 + 0	1 + 1	1 + 2
	Level 3 (2)	2 + 0	2 + 1	2 + 2

**Table 13:** Objective data of the case patient.

General and Anthropometric Data	
Sex	Male
Age	34
Weight (kg)	85
Height (cm)	186
Neck circumference (cm)	46
**Habits**	
Smoking habits	No
Cigarettes per day	-
Years smoking	-
Drinking habits	Occasionally
Grams of alcohol	-
**Diagnosed Conditions**	-
**Prescribed Treatments**	-

**Table 14:** Subjective data of the case patient.

Sleep Time Group	
Hours of sleep	7 h
Minutes until falling asleep	20 min
Prolonged intra-sleep awakenings	Often
**Unrefreshing Sleep Group**	
Feeling of unrefreshing sleep	No
Daytime tiredness	No
Morning dullness	Occasionally
**Complicating Sleep Factors Group**	
Unjustified multiple awakenings	No
Nocturia	Often
Breathless awakenings	No
Reported apneas	Often
**Snores Group**	
Snorer	Yes
High intensity snorer	Yes
Snoring-related awakenings	Occasionally

**Table 15:** Thresholds for the first risk assessment of the case study.

Symbolic Risk		
Level	Case	Level Interpretation
Level 1	IF Risk < 45 THEN Level 1	Non-OSA case
Level 2	IF 45 ≤ Risk < 65 THEN Level 2	Doubtful case
Level 3	IF Risk ≥ 65 THEN Level 3	Possible OSA case
**Statistical Risk**		
Level	Case	Level Interpretation
Level 1	IF Risk < 45 THEN Level 1	Non-OSA case
Level 2	IF 45 ≤ Risk < 60 THEN Level 2	Doubtful case
Level 3	IF Risk ≥ 60 THEN Level 3	Possible OSA case

**Table 16:** Risk assessment of the case study. The colours refer to the recommendations suggested by the system. The green colour indicates a non-OSA case; the orange colour indicates a doubtful case; the red colour indicates a possible OSA case.

	**Statistical Risk**	Risk < 45	45 ≤ Risk < 60	Risk ≥ 60
	**Symbolic Risk**	Risk < 45	45 ≤ Risk < 65	Risk ≥ 65
	Level & Score	Level 1 (0)	Level 2 (1)	Level 3 (2)
	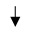			
	**Symbolic Risk**			
**Statistical** **Risk**	Level & Score	Level 1 (0)	Level 2 (1)	Level 3 (2)
	Level 1 (0)	-	X = 1	-
	Level 2 (1)	-	-	-
	Level 3 (2)	-	-	-

**Table 17:** Benchmarking.

Proposals	Efficiency	Scalability	Inference	Learning
Corrado Mencar et al. [37]; Lei Ming Sun et al. [38]; and Jayroop Ramesh et al. [39].	These proposals are based on the use of machine-learning techniques or optimization approaches, which manage uncertainty using a probabilistic approach.	These proposals lack scalability.	The systems rely on statistical inference methods rather than symbolic reasoning for their operation.	These systems incor-porate knowledge in a way that is subsidiary to its classification process.
	=	-	-	=
Ferreira-Santos et al. [40]	The authors use Bayesian network approaches. An implicit management of uncertainty is used, based in the calculation of probabilities.	The system is not scalable, as it is associated with the network model.	Statistical inference is used instead of symbolic reasoning.	The system incorporates knowledge in a way that is subsidiary to the Bayesian network.
	=	-	-	=
C. Zoroglu and S. Turkeli [41]; and J. M. Matthews et al. [42]	The authors use fuzzy inference systems, which manage uncertainty from a non-probablistic point of view.	The systems are not scalable.	The systems use a deductive symbolic reasoning method.	The systems possess a knowledge base associated with the inference engine. It has the capability to incorporate new knowledge.
	=	-	=	=
Casal-Guisande et al. [21]	The authors use several machine-learning algoritms, as well as ANFIS and a heuristic algorithm. The proposed system effectively handles uncertainty from both a probabilistic and non-probabilistic perspective.	The system is scalable, as it is possible to modify the calculation blocks.	The system uses both statistical and symbolic inference approaches, but does not fully formalize a knowledge base.	New knowledge can be easily integrated into the system while it is in use.
	=	=	-	=
Our proposal	The proposed system, based on the use of heteregenous inferential approaches, both statistical and symbolic, manages uncertainty from both a probabilistic and non-probabilistic perspective.	The proposed system is scalable, as it is possible to modify the calculation and inference modules.	The proposed system used both symbolic and statistical inference approaches, with a complete formalization of knowledge.	The system has the capability to model and incorporate new knowledge.

## Data Availability

Not applicable.
